# Starting an Older Adult Lecture for First Semester Nursing Students With a Truth or Myth Game

**DOI:** 10.1093/geroni/igaa057.047

**Published:** 2020-12-16

**Authors:** Lynn Brown, Pao-feng Tsai

**Affiliations:** Auburn University, Auburn, Alabama, United States

## Abstract

False ideas about the physical and psychosocial characteristics of older adults exist in America. It is especially important that nurses are not susceptible to myths and stereotypes as these myths can affect the quality of patient care. For example, some people stereotype older adults as forgetful, disabled, ill, and unable to understand new information. Misconceptions and negative stereotypes are also present in first year nursing students. It is vital that students assess their own attitudes about older adults to form positive attitudes and gain knowledge about aging and health care needs. To achieve this goal, the older adult lecture in a first semester theory and fundamental course begins with a PowerPoint slide presentation asking students to distinguish truths and myths. The truth or myth topics include a) developmental tasks; b) common physiological changes; c) a comparison of delirium, dementia, and depression; and d) addressing health concerns of older adults. Active discussion follows the activity. Seventy to ninety percent of students correctly answered nine of ten questions related to older adult content on the final exam. Considering the increasing number of older adults in the health care setting, nurse educators must dismantle negative stereotypes with creative teaching strategies.

